# Diaphragm Tracking System That Superimposes Planned Diaphragm Contours on Kilovolt Cine Images During Multiple Breath-Hold Volumetric Modulated Arc Therapy for Abdominal Tumors

**DOI:** 10.7759/cureus.67540

**Published:** 2024-08-22

**Authors:** Yuki Nozawa, Takeshi Ohta, Atsuto Katano, Hideomi Yamashita, Keiichi Nakagawa

**Affiliations:** 1 Radiology, University of Tokyo Hospital, Tokyo, JPN; 2 Comprehensive Radiatiion Oncology, University of Tokyo, Tokyo, JPN

**Keywords:** kilovolt projection, drr, abdominal tumors, breath-hold, projection streaming, diaphragm contour, diaphragm tracking

## Abstract

We recently published a phantom validation of our diaphragm tracking system, DiaTrak, on an Elekta linear accelerator with an integrated cone-beam computed tomography (CBCT) unit for multiple breath-hold volumetric modulated arc therapy of abdominal tumors, where the diaphragm position was compared between digitally reconstructed radiography (DRR) and kilovolt (kV) projection streaming images by template matching. In the present report, the visual feedback of the diaphragm position was added to the reported system. DICOM-RT diaphragm contour data were additionally exported from a treatment planning system to the DiaTrak PC. Following phantom localization by registering the CBCT to the planning CT images, a projected diaphragm contour was overlaid on each DRR image, whereas another two projected diaphragm contours were superimposed on each kV projection cine image every 180 ms after shifting ±5 mm (set as breath-hold tolerance) in the craniocaudal direction during gantry rotation. It was visually confirmed that the projected diaphragm surface was observed within the two contour lines on the kV cine window. The diaphragm registration errors of the localized phantom were also calculated based on image cross-correlation between the DRR and the projection cine images every 180 ms. It was found that the mean diaphragm registration error was -0.29 mm with a standard deviation of 0.32 mm during the gantry rotation. In conclusion, a new interface for the 5 mm tolerance check was proposed to provide direct visual feedback, thereby giving a sense of assurance to the attending radiotherapy technologists. The calculated diaphragm registration errors were relatively small compared to the tolerance of 5 mm, and therefore it is considered clinically acceptable.

## Introduction

It was reported that liver tumor and diaphragm motions had high concordance when the distance between the tumor and tracked diaphragm area was small [1-3]. Therefore, we recently published a phantom validation of a diaphragm tracking system, DiaTrak, on an Elekta linear accelerator (linac) with an integrated cone-beam computed tomography (CBCT) unit for multiple breath-hold volumetric modulated arc therapy (VMAT) of abdominal tumors, where diaphragm positions were continuously compared between digitally reconstructed radiography (DRR) and kilovolt (kV) projection streaming images by template matching during VMAT delivery in every 180 ms [4]. The calculated diaphragm registration errors were displayed in every 180 ms on the DiaTrak application window by locating the maximum normalized cross-correlation coefficient while translating the kV projection image in the craniocaudal direction with an increment of 1 mm. This functionality may be appropriate when the DiaTrak system works with an automatic beam gating unit equipped inside a linac system. However, it may not be the best when attending radiotherapy staff would prefer more direct visual feedback so that they can pause the treatment beam any time when the diaphragm position moves out of tolerance. This is still practical because a typical number of breath-hold cycles for 6 MV flattening filter-free (FFF) liver VMAT is six in our facility. The purpose of this paper is to propose a new interface for more direct visual feedback and to provide a sense of treatment assurance for the attending technologists.

## Technical report

**Figure 1:**
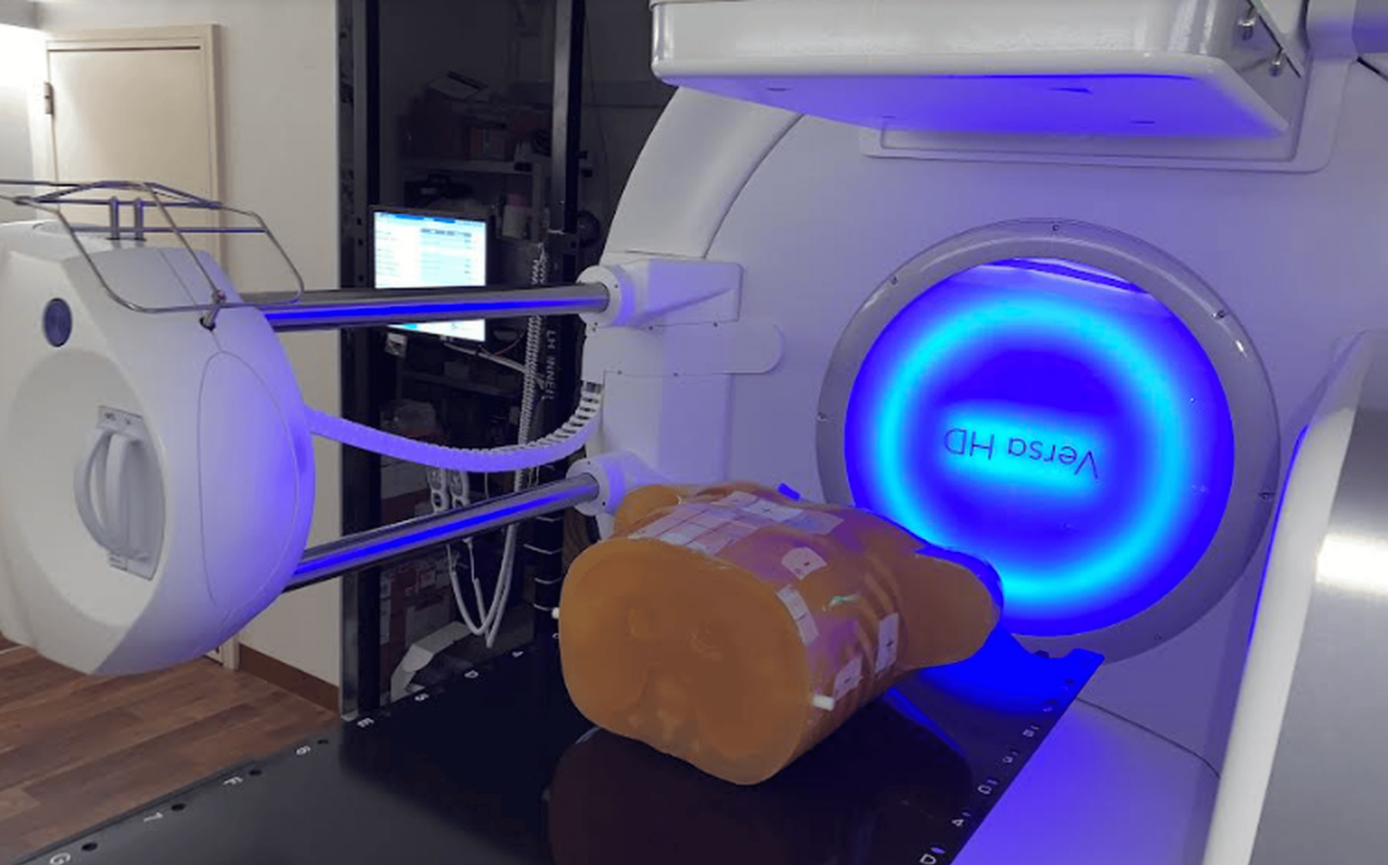
A photograph of a chest phantom placed on a treatment couch. A CBCT scan was performed and compared to a previously acquired planning CT scan to adjust the position of the couch for phantom localization. CBCT: cone-beam computer tomography

Figure 1 shows a photograph of a chest phantom placed on a treatment couch. A CBCT scan was performed and registered to a previously acquired planning CT scan to adjust the position of the couch for the phantom localization using a CBCT unit, X-ray volume imaging (XVI), version 5.0.7 (Elekta AB, Stockholm, Sweden).

The DRR was pre-calculated from the planning CT images at each degree of gantry angle for subsequent real-time template matching, whereas the kV projection images were exported to an external DiaTrak PC through an Elekta’s projection streaming research interface, where the streaming data included kV projection images and kV beam angles. By knowing the kV beam angle, a DRR image with an integer angle can be selected, which is the nearest neighbor to the kV beam angle. The diaphragm registration error was calculated every 180 ms during gantry rotation by comparing the DRR and the kV projection cine images acquired using the CBCT unit.

Prior to the treatment, DICOM-RT diaphragm contour data on each slice were also exported from a treatment planning system to the DiaTrak PC. The abdominal organs bounded by the diaphragm were contoured on each slice, and the resulting three-dimensionally constructed curved surface was defined as the contour of the diaphragm. Subsequently, the contour data on each slice was converted to enclosed volume data, where the intensity of each voxel inside the contours was assigned a value of one, and that outside the contours was assigned a value of zero. Then, the DRR of this contour volume was calculated at each degree of gantry angle, leading to each projection of the contour volume at each gantry angle. By recording the coordinates of pixel values increasing from zero on each line from superior to inferior direction, the projected diaphragm contour line was obtained at each degree of gantry angle.

During gantry rotation, a projected diaphragm contour was overlaid on each DRR image at each gantry angle. Furthermore, two projected contours were superimposed on the kV projection cine image every 180 ms after shifting ±5 mm (set as breath-hold tolerance) in the craniocaudal direction.

The diaphragm registration errors of the localized phantom were also calculated based on image cross-correlation between the DRR and the projection cine images every 180 ms, where the cine image was translated in the craniocaudal direction with an increment of 1 mm. Then, the registration error was given by the translated amount giving the highest cross-correlation. To calculate the registration error more precisely, a parabolic interpolation was performed using three consecutive sample points 1 mm apart, including the maximum correlation. The details of the method for calculating registration errors were described in our previous paper [4].

Video 1 demonstrates a DRR window on the left and a kV cine window on the right, with the template matching results displayed at the center in green after three-point parabolic interpolation every 180 ms during gantry rotation. The image cross-correlation was calculated within the rectangular region of interest (ROI), which is positioned above the image center and includes the diaphragm. On the left DRR window, the projected diaphragm contour was superimposed. On the right kV cine window, the diaphragm contour was shifted ±5 mm in the craniocaudal direction to show the breath-hold tolerance. The maximum voxel intensity outside the ROI on the DRR window was normalized to that inside the ROI in order to provide the highest contrast inside the ROI. The same normalization was performed to the kV cine window as well.

**Video 1:** An application showing template matching results of the phantom between DRR and kV cine images in every 180 ms during gantry rotation. The image cross-correlation was calculated within the rectangular region of interest, which is positioned above the image center and includes the diaphragm. DRR: digitally reconstructed radiography; kV: kilovoltage

**Figure 2:**
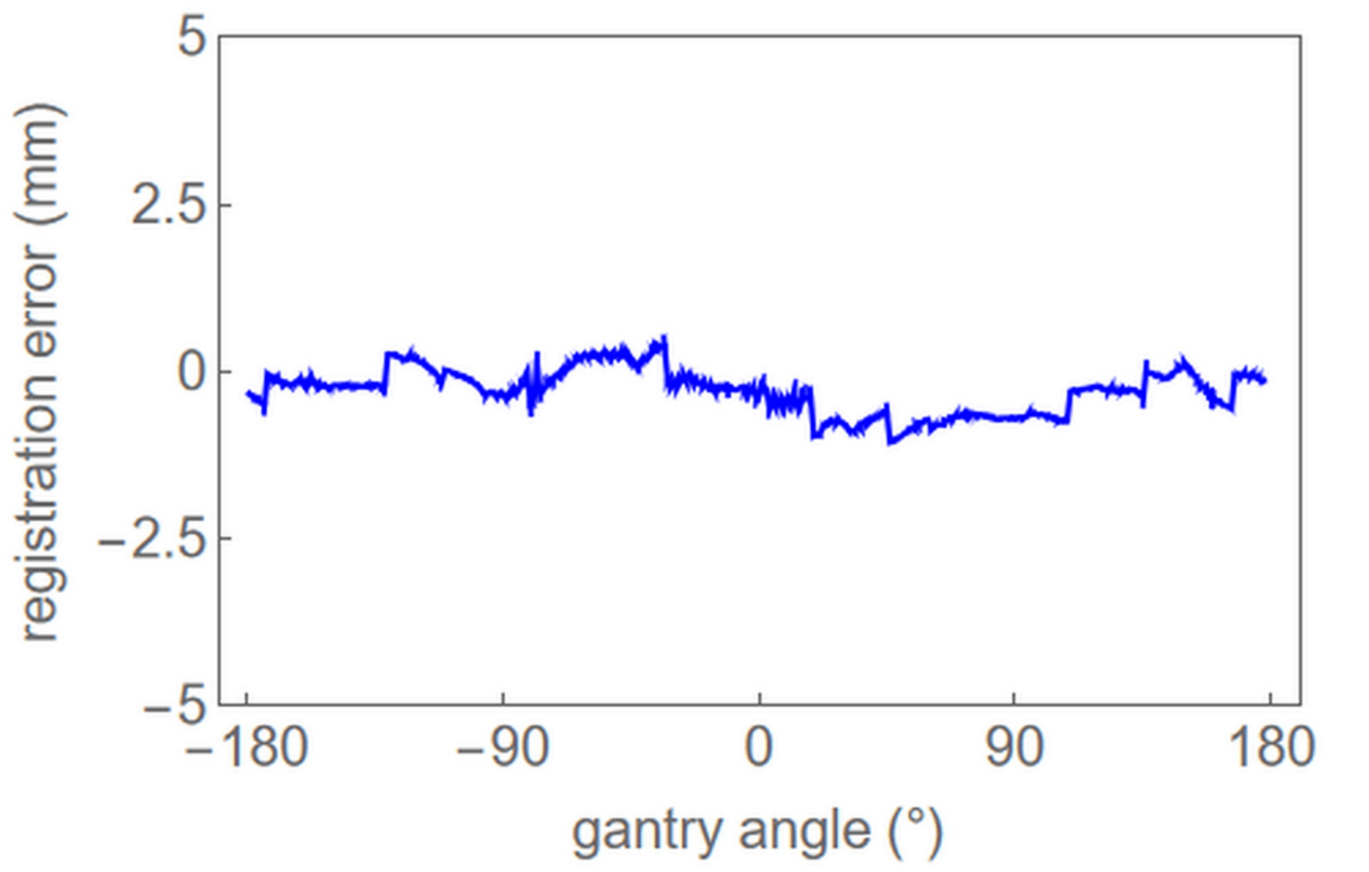
A plot of calculated registration errors using the template matching as a function of the gantry angle.

Figure 2 depicts a plot of registration errors calculated from Video 1 as a function of the gantry angle. The registration errors were calculated as translated amounts giving the maximum cross-correlation followed by three-point parabolic interpolation for more precision. The mean diaphragm registration error over all gantry angles was -0.29 mm with a standard deviation of 0.32 mm.

## Discussion

As shown in Video 1, it is possible to display the tolerances of the diaphragm position of the chest phantom predicted from the DRR in real time at each gantry angle. Direct visual feedback of the diaphragm position allows the attending radiotherapy technologists to evaluate whether the projected diaphragm surface is observed within the two contour lines that indicate the breath-hold tolerance with a sense of treatment assurance.

Since there was no non-rigid deformation in the chest phantom and clear linearity was obtained in the experiment by manually moving the couch at a certain amount [4], this analysis is considered reproducible. Therefore, Figure 2 shows the results of a single measurement performed on a chest phantom with a gantry rotation of 360 degrees.

As shown in Figure 2, the gantry angle dependence of the registration error with the DiaTrak system is sufficiently small compared to the set tolerance of ±5 mm for breath-hold irradiation. It is therefore considered reliable and clinically useful. However, the density and the rigidity of a patient are different from those of the phantom. In this study, the diaphragm displacement in the craniocaudal direction is given by the image cross-correlation between the DRR and the kV cine images within each ROI as described in the Technical Report section. This indicates that the calculated displacement for a non-rigid patient diaphragm is considered as a mean displacement in the craniocaudal direction within the ROI. Further study is required to evaluate the effect of those factors on registration errors by in-treatment patient data.

## Conclusions

A new visual interface for tolerance check has been proposed in the DiaTrak system during multiple breath-hold VMAT delivery, allowing the attending technologists to confirm the diaphragm positions and a sense of treatment assurance. The projected phantom diaphragm surface was observed within the two contour tolerance lines on the kV cine window. The diaphragm registration errors of the localized phantom were also calculated based on image cross-correlation between the DRR and the projection cine images, resulting in a mean diaphragm registration error of -0.29 mm with a standard deviation of 0.32 mm during the gantry rotation. The calculated diaphragm registration errors were relatively small compared to the tolerance of 5 mm, and therefore it is considered clinically acceptable.
